# Supplementary material for: Minimizing features while maintaining performance in data classification problems
Source: PeerJ Comput Sci. 2022 Sep 14;8:e1081. doi: 10.7717/peerj-cs.1081 (PMC9575878; doi:10.7717/peerj-cs.1081)
Supplement: Supplemental Information 2 [file peerj-cs-08-1081-s002.pdf]

A SIMULATION RESULTS FOR DIFFERENT CLASSIFICATION MODELS

Referring to the Simulation Results Section, Figures 11, 12, 13 and 14 present the results of the comparison of RFE, PCLFS, and PCLFS-ext methods for other classification models such as LGBM_C, Decision Tree, RFC and SVM_Linear with highly imbalanced data with 90%:10% rate and a sample size of 1000. As discussed in Sec 3, it is observed that, other than having higher model F1-scores and feature selection correct percentages, PCLFS-ext method also selects a lower number of features for many choices of informative features than the RFE method.

A.1 Light Gradient Boosting (LGBM_C)

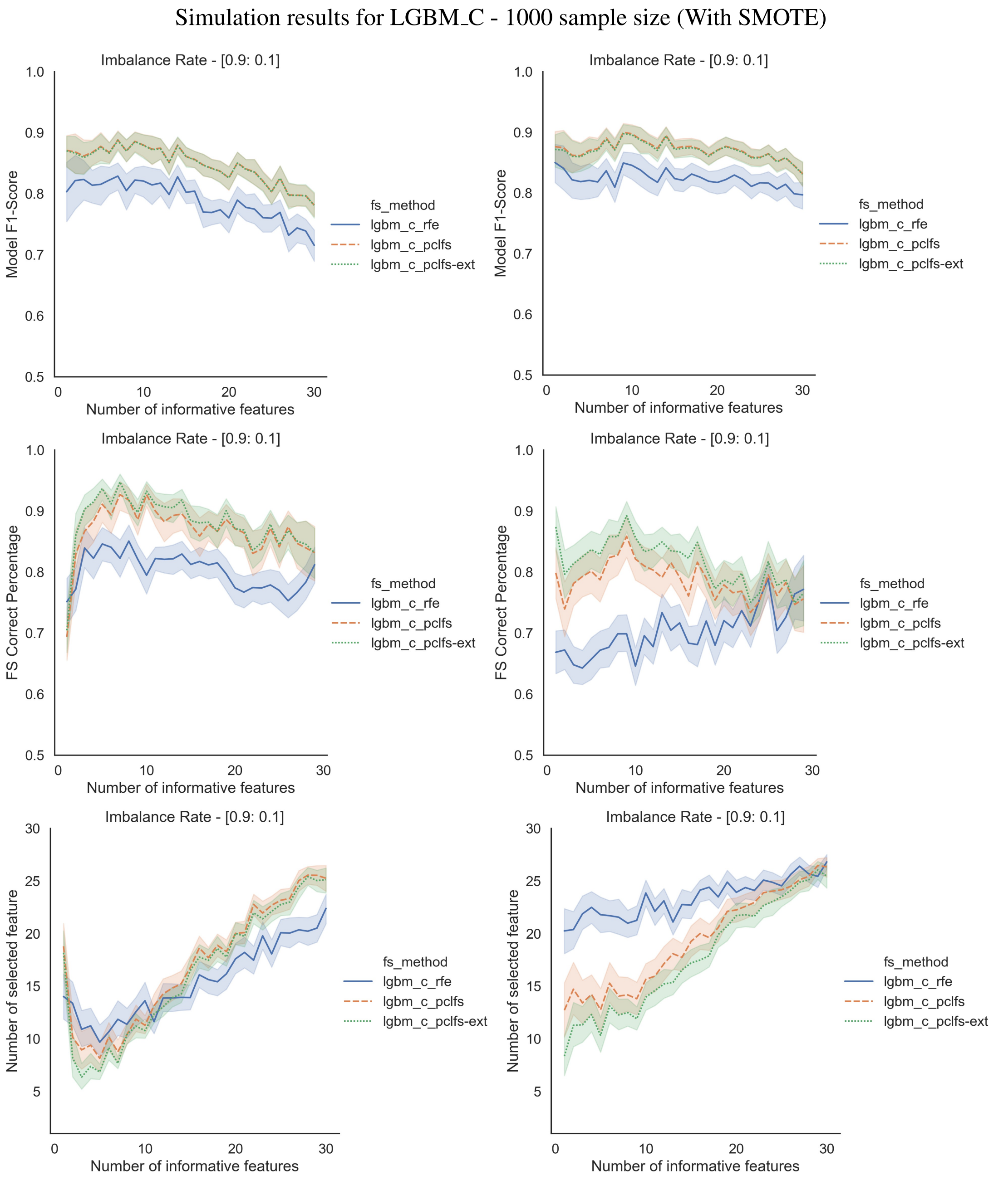

Figure 11. Rows represent final F1-scores, Feature selection correct percentages, and the number of informative selected features, whereas the left-hand side column with original data and right is with SMOTE data for the Lgbm_C classifier with a threshold of 0.0017.

A.2 Decision Trees

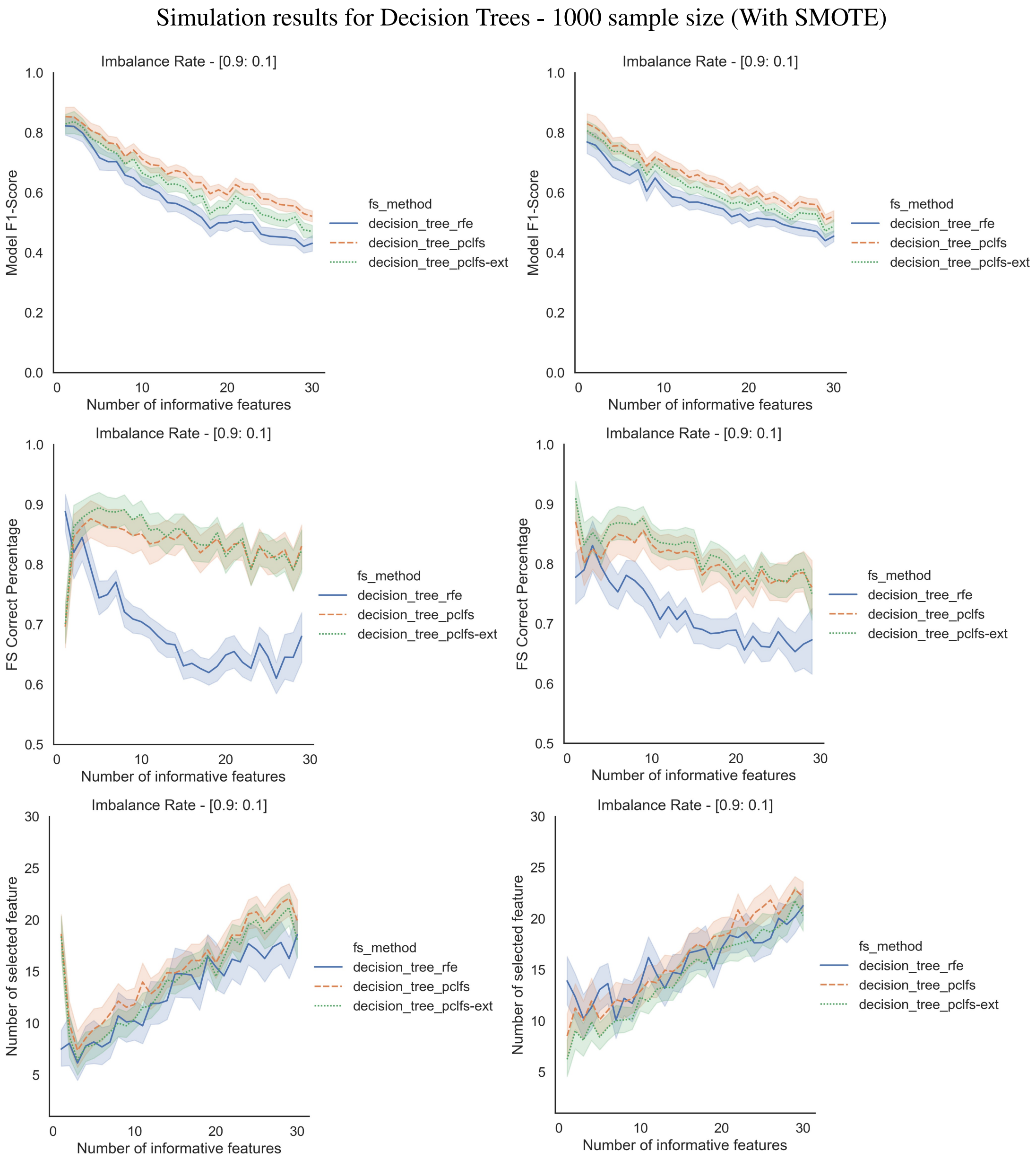

Figure 12. Rows represent final F1-scores, Feature selection correct percentages, and the number of informative selected features, whereas the left-hand side column with original data and right is with SMOTE data for the Decision tree classifier with a threshold of 0.0017.

A.3 Random Forest Classifier (RFC)

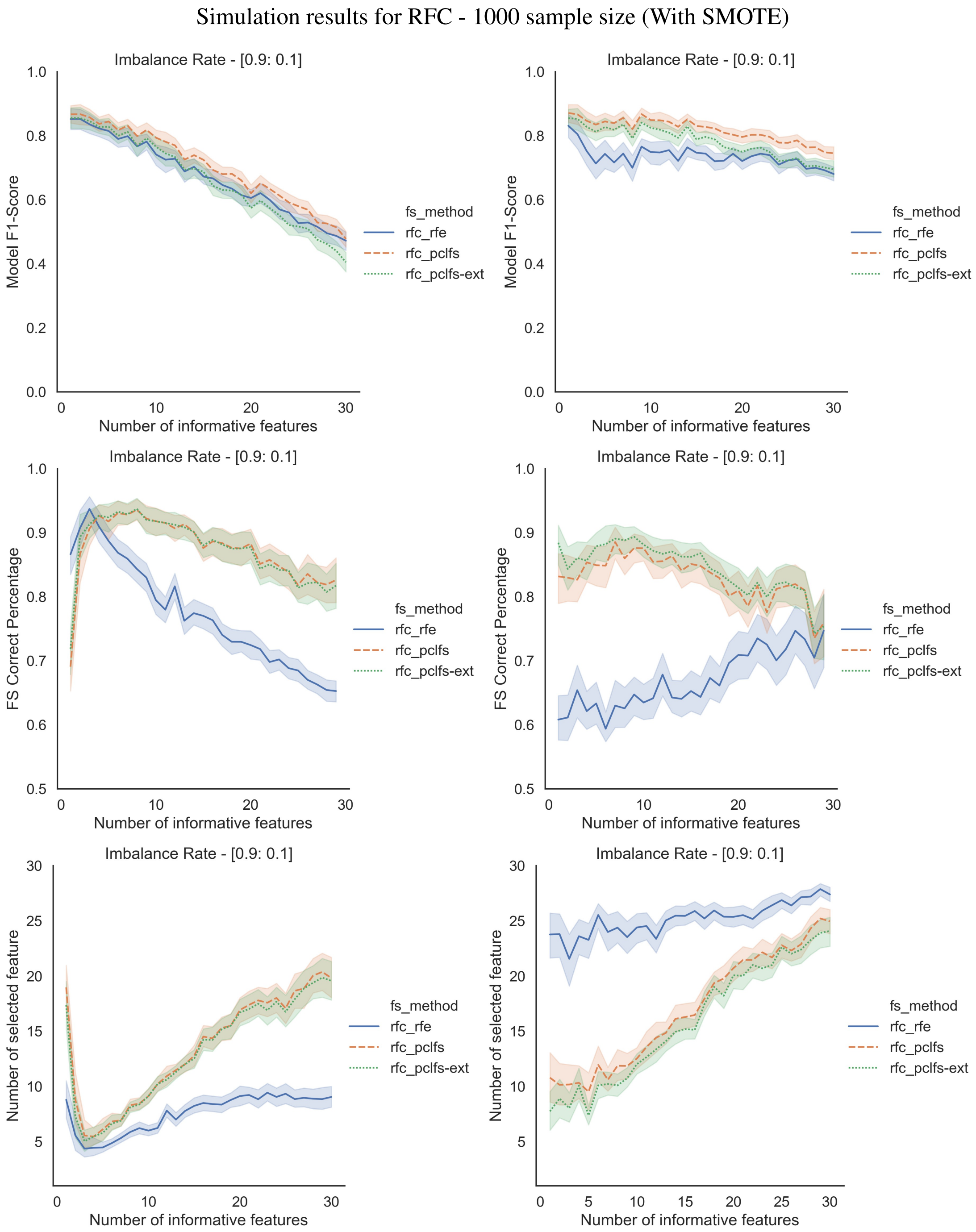

Figure 13. Rows represent final F1-scores, Feature selection correct percentages, and the number of informative selected features, whereas the left-hand side column with original data and right is with SMOTE data for the RFC with a threshold of 0.0017.

A.4 SVM_Linear

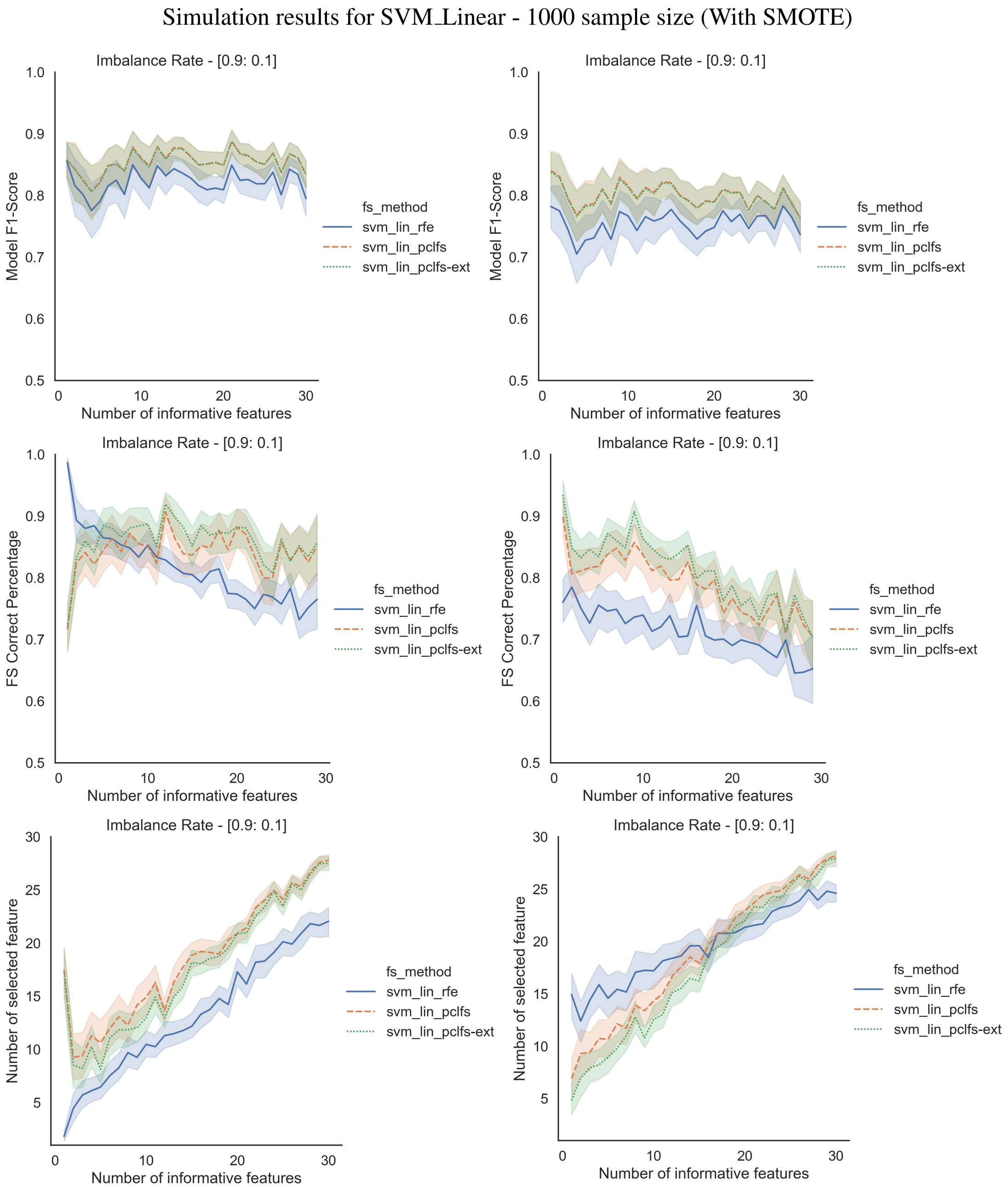

Figure 14. Rows represent final F1-scores, Feature selection correct percentages, and the number of informative selected features, whereas the left-hand side column with original data and right is with SMOTE data for the SVM-linear classifier with a threshold of 0.0017.
